# Outcomes of complex colorectal polyps managed by multi-disciplinary team strategies—a multi-centre observational study

**DOI:** 10.1007/s00384-022-04299-0

**Published:** 2023-02-03

**Authors:** J. Parker, S. Gupta, L. Shenbagaraj, P. Harborne, R. Ramaraj, S. Karandikar, M. Mottershead, J. Barbour, N. Mohammed, M. Lockett, A. Lyons, R. Vega, J. Torkington, S. Dolwani

**Affiliations:** 1 School of Medicine and Cardiff and Vale University Health Board, Cardiff University, Cardiff, UK; 2 Guy’s and St. Thomas’ NHS Foundation Trust, London, UK; 3 United Lincolnshire Hospital Trust, Lincoln, UK; 4 Cardiff and Vale University Health Board, Cardiff, UK; 5 University Hospitals Birmingham Foundation NHS Trust, Birmingham, UK; 6 Gateshead Health NHS Foundation Trust, Gateshead, UK; 7 Leeds Teaching Hospitals NHS Trust and University of Leeds, Leeds, UK; 8 North Bristol NHS Trust, Bristol, UK; 9 University College London Hospitals NHS Foundation Trust, London, UK

**Keywords:** Large or complex colorectal polyp, Multi-disciplinary team management, Decision-making, Outcomes

## Abstract

**Purpose:**

Team management strategies for complex colorectal polyps are recommended by professional guidelines. Multi-disciplinary meetings are used across the UK with limited information regarding their impact. The aim of this multi-centre observational study was to assess procedures and outcomes of patients managed using these approaches.

**Method:**

This was a retrospective, observational study of patients managed by six UK sites. Information was collected regarding procedures and outcomes including length of stay, adverse events, readmissions and cancers.

**Results:**

Two thousand one hundred ninety-two complex polyps in 2109 patients were analysed with increasing referrals annually. Most presented symptomatically and the mean polyp size was 32.1 mm. Primary interventions included endoscopic therapy (75.6%), conservative management (8.3%), colonic resection (8.1%), trans-anal surgery (6.8%) or combined procedures (1.1%). The number of primary colonic resections decreased over the study period without a reciprocal increase in secondary procedures or recurrence. Secondary procedures were required in 7.8%. The median length of stay for endoscopic procedures was 0 days with 77.5% completed as day cases. Median length of stay was 5 days for colonic resections. Overall adverse event and 30-day readmission rates were 9.0% and 3.3% respectively. Malignancy was identified in 8.8%. Benign polyp recurrence occurred in 13.1% with a median follow up of 30.4 months. Screening detected lesions were more likely to undergo bowel resection. Colonic resection was associated with longer stays, higher adverse events and more cancers on final histology.

**Conclusion:**

Multi-disciplinary team management of complex polyps is safe and effective. Standardisation of organisation and quality monitoring is needed to continue positive effects on outcomes and services.

**Supplementary Information:**

The online version contains supplementary material available at 10.1007/s00384-022-04299-0.

## Introduction

Colorectal polyps are often a precursor to malignancy [1] and removal can reduce the incidence of bowel cancer [2]. Increasing detection is likely due to colorectal cancer screening programmes [3], improvements in colonoscopy and increasing awareness of symptoms. The morphological spectrum of colorectal polyps is considerable. The size, morphology, site, access (SMSA) scoring system is validated in determining lesion complexity and difficulty of polypectomy [4]. For those with a higher SMSA level, the decision-making and technical challenges of treatment are significant. With a 10 to 15% risk of containing a focus of cancer [5], accurate lesion and patient assessment is required. Management should be individualised, and options include endoscopic resection, combined procedures, conservative management or surgery including trans-anal approaches and colonic resection. Endoscopic intervention is recommended first line [5], but variability remains in the management of these lesions [6, 7]. Static or increasing use of colonic resection has been reported despite advances in organ preserving techniques [8, 9].

Endorsed by guidelines, multi-disciplinary management meetings for complex colorectal polyps are used across the UK [5]. These meetings are synonymous to tumour boards used in other countries. Effectiveness has been demonstrated elsewhere [10, 11], but understanding of their impact on complex polyp outcomes is limited. The primary aim of this multi-centre observational study was to assess procedures and clinical outcomes of patients managed through these approaches. Other objectives included assessment of referral volume, trends in primary procedures and comparisons between presentation and treatment modality.

## Method

This was a retrospective, observational study of consecutive patients managed by six complex polyp multi-disciplinary team meetings in the UK utilising the STROBE recommendations [12].

### Data collection

Each centre provided prospective lists of patients referred to meetings from commencement for review and assessed until March 2020 at the latest. Data were collected from digital hospital records onto pre-defined spreadsheets.

### Patient and polyp demographics

Data were collected regarding patient and polyp characteristics. Screening patients were diagnosed through colorectal cancer screening programmes. Symptomatic patients included those diagnosed through symptomatic presentations, incidental findings, or through surveillance programmes. Comorbidities were described using the Charlson Comorbidity Index (CCI) [13] and polyp complexity defined by the SMSA scoring system [4].

### Outcomes

Length of stay was the total nights in hospital. Adverse events were classified using the Clavien-Dindo (CD) system [14]. Bleeding controlled during a procedure without additional intervention was not considered an adverse event. Readmission rate was unplanned readmissions related to the polyp procedure within 30 days. Residual or recurrent disease included histologically confirmed lesions at or adjacent to the original excision site identified at follow-up colonoscopy.

### Inclusion and exclusion criteria

Standardised criteria for case selection were used with at least one year follow-up to allow time for surveillance to be performed. Patients with no documentation regarding meeting discussion were excluded. Lesions referred but on assessment were absent or did not meet complexity criteria were also excluded. This included those below 10 mm and without other complexity indicators such as difficult access, recurrence or advanced histology signs. Non-neoplastic pathology, multiple small polyps and polyposis syndromes were excluded. The study focussed on lesions initially assessed as benign so confirmed cancers before intervention were excluded. Patients pending treatment or follow-up were reported but not analysed.

### Statistical analysis and comparisons

Descriptive statistics were performed with unpaired *t* and Mann–Whitney *U* tests for parametric and non-parametric data respectively. Chi-squared was used for categorical data. Comparisons were made between presentation type and colonic resections against organ sparing procedures. Statistical analysis was performed with SPSS version 26 (IBM, Chicago, IL, USA). A *P* value < 0.05 was considered significant.

### Ethics

As a service evaluation, further ethical approval was deemed unnecessary by Cardiff University Research Integrity, Governance and Ethics Team. Local research governance guidance was followed at each site.

## Results

### Patient and polyp demographics

A total of 2749 patients were referred with increasing numbers each year. Exclusion of 640 cases left 2109 patients for analysis (Supplementary materials 1 and 2).

Table 1 summarises patient and polyp characteristics. The mean age was 68.9 years with most presenting symptomatically. There was a male preponderance in all categories and symptomatic patients had a significantly higher CCI. Supplementary material 3 shows characteristics of each centres team structure.

**Table 1:** Patient and polyp characteristics

**PATIENT CHARACTERISTICS**				
	**Total (*n* = 2109)**	**Screening (*n* = 749)**	**Symptomatic (*n* = 1360)**	***P* value**
Age (years)	68.9 (23 to 97)	67.5 (50 to 78)	69.7 (23 to 97)	< 0.001
Female	832 (39.5%)	247(33.0%)	585(43.0%)	< 0.001
Male	1277 (60.5%)	502 (67.0%)	775(57.0%)	
CCI	3.5 (0 to 12)	3.1 (0 to 8)	3.7 (0 to 12)	< 0.001

**POLYP CHARACTERISTICS**				
	**Total (*n* = 2192)**	**Screening (*n* = 758)**	**Symptomatic (*n* = 1434)**	***P* value**
Polyp size (mm)*	32.1 (2 to 180)	33.6 (2 to 120)	31.4 (3 to 180)	0.005
Polyp morphology				
Flat	829 (37.8%)	238 (31.4%)	591 (41.2%)	
Sessile	1130 (51.6%)	455 (60.0%)	675 (47.1%)	
Pedunculated	228 (10.4%)	60 (7.9%)	168 (11.7%)	
Missing	5 (0.2%)	5 (0.7%)	0	
Polyp location				
Right	980 (44.7%)	340 (44.9%)	640 (44.6%)	0.920
Left	1212 (55.3%)	418 (55.1%)	794 (55.4%)	
Polyp access				
Difficult	1024 (46.7%)	199 (26.3%)	825 (57.5%)	
Easy	1168 (53.3%)	559 (73.7%)	609 (42.5%)	
SMSA level				
4	971 (44.3%)	324 (42.7%)	647 (45.1%)	0.401
3	788 (35.9%)	278 (36.7%)	510 (35.6%)	
2	420 (19.2%)	144 (19.0%)	276 (19.2%)	0.002
1	8 (0.4%)	7 (0.9%)	1 (0.1%)	
Missing	5 (0.2%)	5 (0.7%)	0	
Previously treated polyp				
Yes	117 (5.3%)	49 (6.5%)	68 (4.7%)	0.088
No	2075 (94.7%)	709 (93.5%)	1366 (95.3%)	
Pre procedure histology				
Biopsy not done	1050 (47.9%)	233 (30.7%)	817 (57%)	
Adenoma, LGD	896 (40.9%)	415 (54.8%)	481 (33.5%)	0.001
Adenoma, HGD	183 (8.4%)	83 (11.0%)	100 (7%)	
Serrated	40 (1.8%)	13 (1.4%)	7 (2.0%)	
Hyperplastic	20 (0.9%)	11 (1.7%)	29 (0.5%)	
Normal mucosa	3 (0.1%)	3 (0.4%)	0	
Further assessment endoscopy				0.417
Yes	227 (10.4%)	84 (11.1%)	143 (10.0%)	
No	1965 (89.6%)	674 (88.9%)	1291 (90.0%)	

Age, CCI and polyp size are given as mean and range. The remaining values are given as number and (%) to one decimal place. Unpaired *t* tests are used for continuous variables and chi-squared tests for categorical data

^*^Missing data, *n* = 1

There were 2192 complex colorectal polyps identified in the 2109 patients. Mean size was 32.1 mm and most were SMSA level 4 (44.3%). A pre-intervention biopsy was documented in 52.1% and histology showed high grade dysplasia (HGD) in 16.0% of these.

There was no difference in the number of SMSA level 3 and 4 lesions (*P* = 0.401), polyp location (*P* = 0.920) or previous treatment attempts (*P* = 0.088) between screening and symptomatic groups. Screen detected polyps were larger (33.6 mm vs 31.4 mm) and had more lesions with HGD (11% vs 7%).

### Procedures

**Fig. 1:**
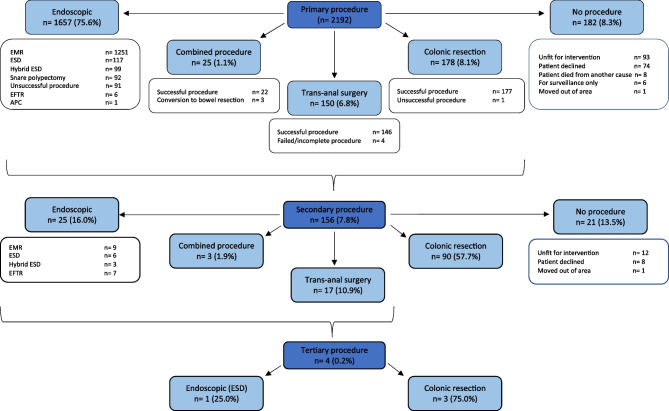
Flow diagram of primary, secondary and tertiary procedures

A total of 2149 procedures were performed on 2192 lesions (Fig. 1). Of these, 2010 were primary procedures with the remainder being secondary (*n* = 135) or tertiary interventions (*n* = 4).

### Primary procedure

Primary endoscopic therapy was performed in 1657 (75.6%) polyps. Surgical procedures were performed in 14.9% including trans-anal surgery (6.8%) or colonic resection (8.1%). Combined endoscopic-surgical procedures and conservative management were used in 1.1% and 8.3% respectively. Reasons for no intervention were mostly due to patients being unfit (51.1%). Other reasons included patients declining treatment (40.7%), opting for surveillance only (3.3%), dying from another cause before treatment (4.4%) or moving out of area (0.5%).

More primary colonic resections were performed in the screening cohort (16% vs 4.7%, *P* < 0.001). Patients undergoing resection were similar in age (68.3 vs 68.4, *P* = 0.862) and gender (59.7% vs 60.6% males, *P* = 0.811) compared to those with organ preservation. Polyps were larger (38.6 mm vs 31.8 mm, *P* < 0.001) in those treated by resection with more right (68.5% vs 41.9%, *P* < 0.001) and SMSA level 3 or 4 lesions (88.2% vs 79.6%, *P* = 0.006). There were more adenomas with pre-intervention HGD in the resection group (23.2% vs 6.2%, *P* < 0.001).

### Secondary and tertiary procedures

Secondary procedures were advised in 156 lesions (7.8%). Indications included unsuccessful primary intervention (38.5%), suspicion of cancer during procedure (23.1%), recurrence (22.4%) or cancer on final histology (16%). Of these, 21 did not have a secondary procedure mostly due to the patient being unfit (57.1%). The commonest secondary procedure was colonic resection (57.7%). Endoscopic management was performed in 16.0% with trans-anal and combined procedures in 10.9% and 1.9% respectively.

Four polyps required a third procedure. Three were due to recurrence and one for cancer detected on final histology. Despite more primary resections in the screening cohort, there was no difference in further procedures between the two presentations (*P* = 0.941).

### Change in recommended procedures over time

**Fig. 2:**
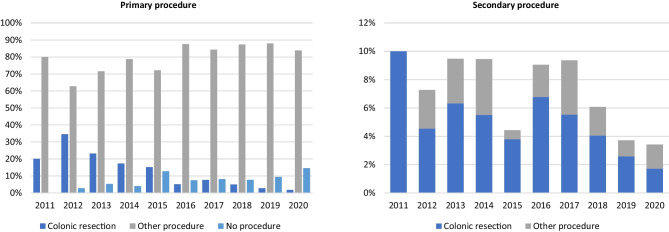
Change in procedures over time

**Fig. 3:**
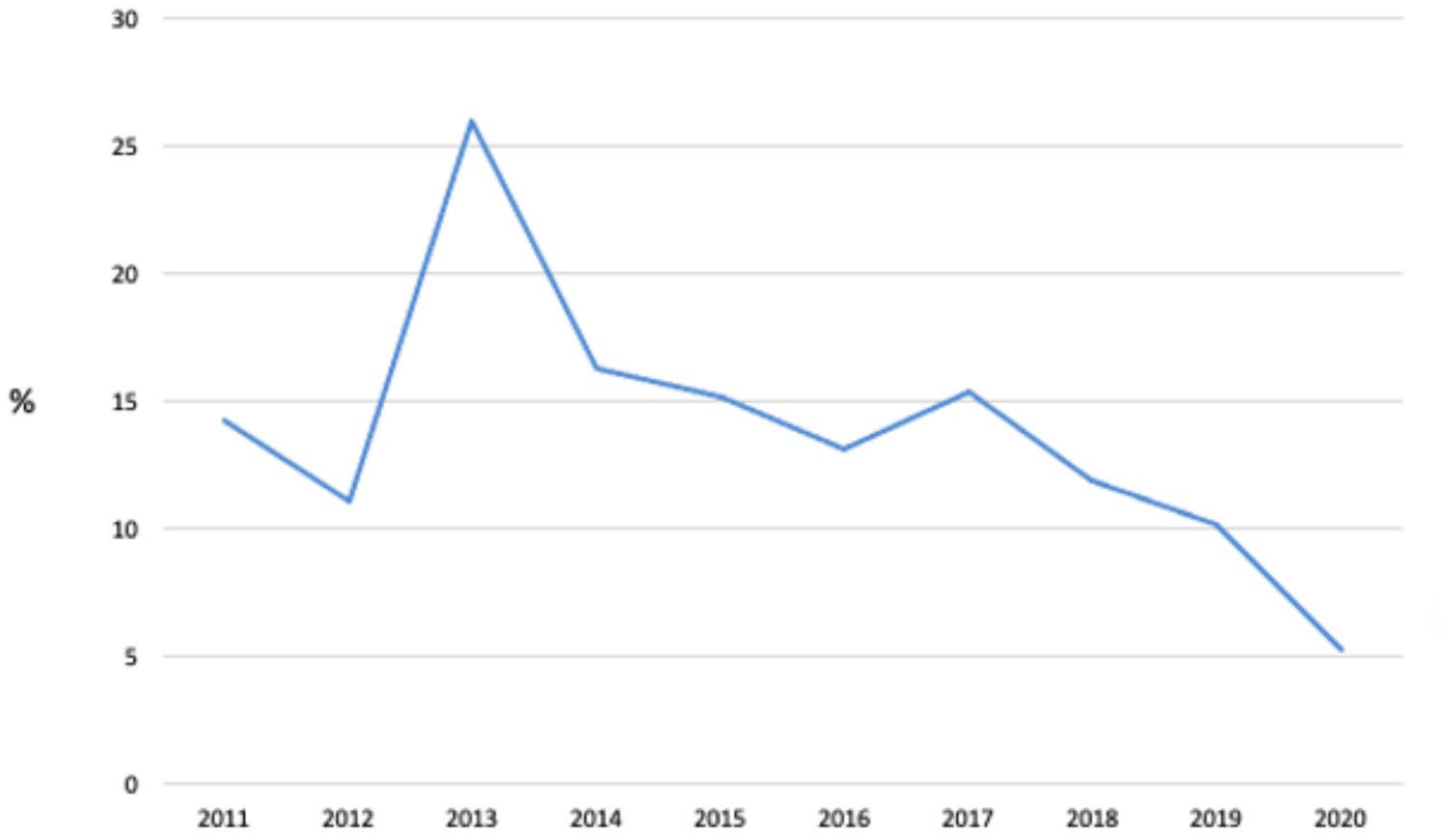
Change in recurrence rates over time

The proportion of primary colonic resections fell from 34.6% in 2012 to 1.7% in 2020 with organ preserving procedures or conservative management having an increasing role (Fig. 2). Over the same time, the use of organ preserving procedures increased from 62.7 to 83.8%. More patients were managed conservatively with 2.7% in 2012 compared to 14.5% in 2020. There was no reciprocal increase in secondary procedures or recurrences as a result of the increasing use of primary organ preserving procedures (Figs. 2 and 3).

### Outcomes

#### Length of stay, adverse events and 30-day readmissions

Most procedures were day cases with a longer length of stay for colonic resections (*P* < 0.001). Adverse events were identified in 9.0% (Table 2) with rates being similar for endoscopic (5.5%), combined (7.1%) and trans-anal procedures (7.2%). Rectal bleeding was the commonest adverse event after endoscopic procedures (3.3%), followed by perforation (0.8%) and post polypectomy syndrome (PPS) (0.7%). Management of bleeding was predominantly conservative (63.6%). A minority required endoscopic intervention (21.8%), transfusion (7.3%), bowel resection (5.5%) or interventional radiology (1.8%). Most perforations occurred in left sided lesions (64.3%) and were managed with antibiotics or surgical intervention in 78.6% and 21.4% respectively.

**Table 2:** Length of stay, adverse events and 30-day readmissions

	**TOTAL** **(*N* = 2149)**	**ENDOSCOPY** **(*N* = 1683)**	**COMBINED PROCEDURE** **(*N* = 28)**	**TRANS-ANAL SURGERY** **(*N* = 167)**	**COLONIC RESECTION** **(*N* = 271)**	***P* VALUE**
**LENGTH OF STAY**	0 (0 to 1)	0 (0 to 0)	2 (2 to 3)	1 (1 to 2)	5 (4 to 8)	*P* < 0.001
**TOTAL ADVERSE EVENTS**	193 (9.0%)	93 (5.5%)	2 (7.1%)	12 (7.2%)	86 (31.7%)	*P* < 0.001
CD 1	65 (33.7%)	45 (48.4%)	2 (100%)	5 (41.7%)	13 (15.1%)	
CD 2	70 (36.3%)	27 (29.0%)	0	4 (33.3%)	39 (45.3%)	
CD 3	32 (16.6%)	15 (16.1%)	0	2 (16.7%)	15 (17.4%)	
CD 4	23 (11.9%)	6 (6.5%)	0	1 (8.3.%)	16 (18.6%)	
CD 5	3 (1.5%)	0	0	0	3 (3.5%)	
**30-DAY READMISSION**	70 (3.3%)	55 (3.3%)	0	2 (1.2%)	13 (4.8%)	*P* = 0.127

Results are described for the total number of procedures performed (*n* = 2149). Figures are given as median (interquartile range) for length of stay. The remaining values are given as number and (%) to one decimal place. *P* values are given for comparisons between colonic resections and all other organ preserving procedures using a Mann–Whitney *U* test for length of stay and chi-squared tests for adverse events and readmissions. A complete overview of adverse events and reasons for 30-day readmissions can be viewed in Supplementary material 4

There were significantly more adverse events for colonic resections (31.7%). The commonest was anastomotic leak (19.8%) which occurred in 11 left and 6 right sided resections. Four were managed conservatively and surgical intervention was required in 13. Wound infection (15.1%), respiratory tract infection (11.6%) and ileus (11.6%) were other frequent adverse events. All three 30-day mortalities occurred in those undergoing colonic resection.

Thirty-day procedure-related readmission was 3.3%. Readmission after colonic resection (4.8%) was higher than endoscopic (3.3%) and trans-anal procedures (1.2%) but not significantly (*P* = 0.127). The commonest readmission reason was rectal bleeding after endoscopic or trans-anal procedures.

#### Final histology

Of the 1989 removed lesions, malignancy was found in 8.8%. Malignancy was significantly higher in the screening cohort (12% vs 7%, *P* < 0.001) and in those having primary colonic resection (26% vs 7%, *P* < 0.001). Of those with HGD on biopsy, 34.4% were identified as cancer on final histology compared to 8.3% with LGD (Supplementary material 5).

Of the cancers, 45.1% had been managed with primary resection. Completion colonic resection was recommended in 14.3% of those treated with organ preservation and 40.6% underwent surveillance only. Seven (9.9%) of these had benign recurrence with four treated during surveillance endoscopy. Three (4.2%) required further procedures with trans-anal surgery (*n* = 3) or colonic resection (*n* = 1).

#### Residual or recurrent disease

The median duration of follow up was 30.3 months (IQR 32.8 to 81.8 months). Of the 2192 lesions, 618 were categorised as not requiring surveillance. Of the remaining 1574, 1209 (76.8%) had a colonoscopy during follow up. Benign recurrence was identified in 13.1% (*n* = 158). Most patients had one episode (*n* = 116) with two or more recurrences in 42 patients. There was no difference in recurrence between screening and symptomatic cohorts (12.8% vs 13.2%, *P* = 0.827). Of the 214 total recurrence episodes, 82.2% were managed at the time of surveillance. Additional procedures were required in 38 (17.8%). Figure 3 demonstrates the reduction in recurrence rates over the study period.

#### Colonic resection

Colonic resection was required in 280 patients. Most were the recommended primary intervention (63.6%). Other indications included unsuccessful primary procedures (10.7%), cancer suspected during treatment (9.3%), cancer on final histology (8.9%) and recurrence (5%). Of the 26 lesions where cancer was suspected during treatment, malignancy was confirmed in 25. Colonic resection was required for adverse events in 2.5% (*n* = 7) (Supplementary material 6).

#### Procedures and outcomes for rectal lesions

There were 642 (29.3%) rectal lesions and endoscopy was the commonest primary procedure (66.8%) Trans-anal procedures were performed in 22.7%, conservative management in 8.3% and colonic resection in 2.2%. Secondary procedures were required in 7% which were mostly colonic resection (51.2%) but also included trans-anal surgery or endoscopy (14.6%). There were no resections performed for adverse events. At the time of follow up, 29.7% of patients with rectal lesions treated surgically still had a stoma.

## Discussion and conclusions

This is the first multi-centre study of team approaches for complex colorectal polyps and demonstrates the delivery of appropriate management with good outcomes. As the case volume is rising and early detection improving, their use may be of increasing importance.

Organ preserving techniques were the primary treatment for most lesions. Primary surgery rate may reflect optimal decision-making, but the standard is not established [5]. Our overall (8.1%) and 2019 (2.7%) primary surgical resection rate is lower than reported (21.7%) [6]. Secondary management (7.8%) was also lower than previous studies by Lee (16.1%) [6] and Dattani (13.2%) [7]. This reduction conflicts the increasing or stable rates reported in American and European studies [8, 9]. Tumour boards in America are analogous to multi-disciplinary team approaches [15], but are not standard practice for complex polyps. Their utilisation in the UK may explain the reduction in colonic resections and have implications for practice standards of professional guidelines [5]. We acknowledge that ongoing developments in advanced endoscopy may confound the observed reduction in colonic resections despite this not having influenced other countries [8, 9]. It also does not explain the increasing utilisation of conservative management seen in this study.

Contrary to previous evidence [7], screening detected polyps were more likely to have primary colonic resection. Some may have been anticipated cancers highlighting one limitation of retrospective data collection. Time allocation for screening lists and more experienced endoscopists may result in lesions being treated without referral to meetings. This could explain the higher number of larger lesions and those with HGD in screening presentations. The lower CCI in screening patients may reflect individual motivation regarding healthcare and mean that surgical treatment is a viable option compared to the comorbid.

The perceived correlation between HGD and cancer on final histology [7] could result in surgery being recommended. Only 34.4% of lesions with pre-intervention HGD were proven to contain cancer, similar to that reported by Dattani (37.5%) [7]. Of lesions with HGD treated with resection, the majority (57.1%) were ultimately found to be benign. Biopsies can create diagnostic uncertainty through sampling error, burden pathology services and compromise endoscopic therapy [16]. Identifying malignant features by optical polyp characterisation is vital for decision-making [17] and the European Society of Gastroenterology now recommend a core curriculum to improve this [18].This can be challenging [19], but quality imaging and training allows final decisions to be made later by those with expertise in this field.

Endoscopic treatment has fewer adverse events, shorter stays and lower costs [20–22] and the safety of procedures in our study being comparable. Post polypectomy bleeding (3.3%) was the commonest adverse event with similar rates reported by Moss (2.9%) and Buchner (7.2%) [16, 23]. Perforation was low (0.8%) and within standards set by guidelines [5]. The thinner right colonic wall may explain the higher resection rates in this group. Most perforations reported in our series were located on the left and managed conservatively. Despite colonic resection offering the security of complete lesion removal, it is overtreatment for most and associated with longer stays and more adverse events. A systematic review of surgical resections for benign polyps reported adverse event and mortality rates of 24% and 0.7% respectively [24]. Our adverse events (31.7%) including a leak rate of 19.8% and mortality of 1.1% are similar and reiterates the greater risks of resection.

Dattani reported a 10.7% risk of cancer in their study of significant polyps [7]. Our cancer rate was 8.8%. Most were managed without completion resection and supports the safety of such management in selected patients. For malignant lesions, survival and recurrence is not adversely affected by endoscopic therapy initially [25] and completion bowel resection may not be superior [26]. Our benign recurrence rate of 13.1% was acceptable. A meta-analysis in 2014 reported recurrence in 15% [27] with more recent evidence quoting 10.8% for large, non-pedunculated polyps [28].

Study limitations include the retrospective design and absence of a control group. A comparator group was considered when designing the study but found not to be pragmatic. Heterogeneity between centres without a meeting could have been misleading. Data collection preceding the introduction of meetings would also have been difficult with limited digital records and challenges in identifying a comparative cohort. Prospective data collection before and after meeting introduction could have been performed but would require considerable time to achieve. All efforts were made to thoroughly assess and record data, but there could be missed adverse events, readmissions and surveillance procedures. Variability between team structure is also a confounder and possibly impacts both the decisions made and outcomes. Despite this, our study provides real world data that should reflect current clinical practice across the UK and outcomes for patients with complex colorectal polyps. We advocate prospective data collection, audit and comparison to key performance indicators ideally on a national scale, to ensure the ongoing effectiveness of polyp meetings.

There may be further benefits of team decision-making. It can improve capacity by modifying management, improving patient preparation and allocating cases to those with expertise [29]. Benefits in clinician education and confidence in choosing organ preserving techniques may result from involvement with meetings. With increasing referrals, ensuring efficiency and appropriate utilisation of polyp meetings is required. Standardised referral criteria and completed proformas [30] are recommended to facilitate efficiency and uniformity. Evaluation of economic impact would also be valuable. Given the spectrum of options for complex polyps and their risks, the patient’s voice is crucial and team management should advocate shared decision-making, with research regarding patient reported outcomes also required.

This data may guide key performance indicators for complex colorectal polyp treatment. The reduction in primary surgery over time suggests that team management of complex polyps contributes to the improvement of clinical outcomes. This effect may be due to a combination of group decision-making, clinical expertise, access to a full range of therapeutic modalities and optimisation of service provision.

### Supplementary Information

Below is the link to the electronic supplementary material.

Supplementary file1 Referrals to complex polyp meetings per year (DOCX 33 KB)

Supplementary file2 Exclusion classifications (DOCX 26 KB)

Supplementary file3 Team characteristics and referrals (DOCX 30 KB)

Supplementary file4 Complications and reasons for 30-day readmissions (DOCX 43 KB)

Supplementary file5 Final histology (DOCX 30 KB)

Supplementary file6 Characteristics of colonic resections (DOCX 31 KB)

## Data Availability

Data is available on request to the lead (JP) and senior (SD) authors.
