# Supplementary material for: Outcomes of complex colorectal polyps managed by multi-disciplinary team strategies—a multi-centre observational study
Source: Int J Colorectal Dis. 2023 Feb 3;38(1):28. doi: 10.1007/s00384-022-04299-0 (PMC9898359; doi:10.1007/s00384-022-04299-0)
Supplement: Supplementary file 1 — Supplementary file1 Referrals to complex polyp meetings per year (DOCX 33 KB) [file 384_2022_4299_MOESM1_ESM.docx]

## SUPPLEMENTARY MATERIAL 1 – Referrals to complex polyp meetings per year

Each bar represents referrals to individual meetings. Numbers are given as total patient referrals each year before exclusions. The dotted line indicates an incomplete year of data as collection ceased in March 2020 at the latest.
