# Supplementary material for: Outcomes of complex colorectal polyps managed by multi-disciplinary team strategies—a multi-centre observational study
Source: Int J Colorectal Dis. 2023 Feb 3;38(1):28. doi: 10.1007/s00384-022-04299-0 (PMC9898359; doi:10.1007/s00384-022-04299-0)
Supplement: Supplementary file 2 — Supplementary file2 Exclusion classifications (DOCX 26 KB) [file 384_2022_4299_MOESM2_ESM.docx]

| Reason for exclusion | Number of patients |
| --- | --- |
| Other pathology or simple polyp found on assessment * | 175 (27.3%) |
| Redirected to cancer meeting for management | 143 (22.3%) |
| Less than 1 year follow-up after primary procedure | 92 (14.4%) |
| Complex polyp not found on assessment ** | 66 (10.3%) |
| Multiple small polyps or polyposis syndrome identified | 59 (9.2%) |
| No documented discussion by complex polyp meeting | 53 (8.3%) |
| Data unavailable | 33 (5.2%) |
| Awaiting management | 19 (3.0%) |
| Total | **640** |

## SUPPLEMENTARY MATERIAL 2 – Exclusion classifications

Figures are given as number of patient and (%) to one decimal place

* Simple polyps were lesions found to be less than 10mm in size with no other high-risk features (such as high-grade dysplasia, recurrent lesions or difficult access) when assessed by the complex polyp meeting

** Most cases were due to lesions detected on other investigations (such as CT colonogram) and not identifiable at endoscopy
