# Supplementary material for: Outcomes of complex colorectal polyps managed by multi-disciplinary team strategies—a multi-centre observational study
Source: Int J Colorectal Dis. 2023 Feb 3;38(1):28. doi: 10.1007/s00384-022-04299-0 (PMC9898359; doi:10.1007/s00384-022-04299-0)
Supplement: Supplementary file 3 — Supplementary file3 Team characteristics and referrals (DOCX 30 KB) [file 384_2022_4299_MOESM3_ESM.docx]

| MDT | Participants | Referral criteria | Source and method of referral | Data collection | Total referrals |
| --- | --- | --- | --- | --- | --- |
| 1 | Gastroenterologist  Colorectal surgeon  Pathologist  Team coordinator  Gastro and surgical trainees  Clinicians from other sites | No agreed criteria  Photos and/or videos required | Own hospital, others within and outside of trust  Email, telephone, face to face conversation  Accepted from consultants, BCSP | Dec-17 to Mar-20  (28 months) | 317 |
| 2 | Gastroenterologist  Colorectal surgeon  Pathologist  Radiologist  Specialist colorectal nurse  Team coordinator  Gastro and surgical trainees  Colorectal oncologists  Clinical trial research nurse | Local guidelines:  Polyps > 6mm  Photos and/or videos required | Own hospital, others within and outside of trust, GP referral centres  Specific complex polyp team proforma  Accepted from registrars, consultants, specialist gastro or colorectal nurses, nurse endoscopists, BCSP | Feb-14 to Mar-20  (73 months) | 527 |
| 3a | Gastroenterologist  Colorectal surgeon  Specialist colorectal nurse  Team coordinator  Gastro trainees | Local guidelines:   1. Laterally spreading tumour (LST) > 2cm regardless of site 2. Right sessile or flat elevated polyp > 2cm 3. Left sessile or flat elevated polyp > 4cm 4. Significant residual or recurrent polyps on scars ≥ 10mm 5. Polyps with difficult access 6. Other (e.g. large pedunculated polyps > 4cm)   Photos and/or videos required | Own hospital, others within trust  Specific complex polyp team proforma  Accepted from junior doctors, registrars, consultants, specialist gastro or colorectal nurses, nurse endoscopists | Jan-15 to Feb-20  (61 months) | 415 |
| 3b | Gastroenterologist  Colorectal surgeon  Pathologist  Radiologist  Specialist colorectal nurse  Team coordinator  Gastro trainees | Local guidelines as 3a  Photos and/or videos required | Own hospital, others within and outside of trust, national referrals  Specific complex polyp team proforma  Accepted from junior doctors, registrars, consultants, specialist gastro or colorectal nurses, nurse endoscopists, BCSPs | Nov-11 to Jul-18  (80 months) | 683 |
| 4 | Gastroenterologist  Colorectal surgeon  Radiologist  Specialist gastro nurse  Nurse endoscopist  Team coordinator  Clinicians from other sites | No definite criteria agreed  Photos required | Own hospital, others outside of trust  Formal letter  Accepted from consultants, specialist gastro or colorectal nurses, nurse endoscopists, BCSP | Mar-14 to Mar-20  (72 months) | 173 |
| 5 | Gastroenterologist  Colorectal surgeon  Specialist gastro and colorectal nurses  Nurse endoscopist  Team coordinator  Gastro and surgical trainees  Endoscopy admin staff | Local guidelines:  Polyps >2cm  Photos required | Own hospital, others within and outside of trust  Specific complex polyp team proforma  Accepted from registrars, consultants, specialist gastro or colorectal nurses, nurse endoscopists, BCSP | Dec-17 to Mar-20  (27 months) | 364 |
| 6 | Gastroenterologist  Nurse endoscopist  Gastro and surgical trainees  Booking team member | No definite criteria agreed  Photos preferred | Own hospital  Email, electronic referral  Accepted from junior doctors, registrars, consultants, specialist gastro or colorectal, nurse endoscopists, BCSP | Oct-18 to Mar-20  (17 months) | 270 |
|  |  |  | **Total** | **358 months** | **2749 patients** |

## SUPPLEMENTARY MATERIAL 3 – Team characteristics and referrals

Supplementary material 3 shows each meeting’s characteristics. All occurred weekly or fortnightly and had both advanced therapeutic endoscopy and surgical techniques available. Site 3 had separate symptomatic (3a) and screening (3b) meetings. Three meetings did not have agreed referral criteria and there was variability in team composition and referral pathways across sites.
