# Supplementary material for: Outcomes of complex colorectal polyps managed by multi-disciplinary team strategies—a multi-centre observational study
Source: Int J Colorectal Dis. 2023 Feb 3;38(1):28. doi: 10.1007/s00384-022-04299-0 (PMC9898359; doi:10.1007/s00384-022-04299-0)
Supplement: Supplementary file 4 — Supplementary file4 Complications and reasons for 30-day readmissions (DOCX 43 KB) [file 384_2022_4299_MOESM4_ESM.docx]

| COMPLICATIONS | | | | | | | | | | | | | | | | | |
| --- | --- | --- | --- | --- | --- | --- | --- | --- | --- | --- | --- | --- | --- | --- | --- | --- | --- |
|  | **Anti-**  **biotics** | **Anti-**  **coag** | **Antibiotics**  **+**  **radiology drain** | **Antibiotics**  **+**  **theatre debridement** | **Conservative** | **Endoscopic**  **intervention** | **EUA and haemostasis** | **IR** | **ICU admission** | **IV fluids** | **Missing** | **NG** | **Colonic resection** | **Surgical washout** | **Temporary catheter** | **Transfusion** | **Total** |
| Endoscopy | **21** | **0** | **0** | **0** | **44** | **12** | **0** | **1** | **0** | **2** | **0** | **1** | **7** | **1** | **0** | **4** | **93** |
| AKI | - | - | - | - | - | - | - | - | - | 1 | - | - | - | - | - | - | 1 |
| Bleeding –  Intra-abdominal | - | - | - | - | - | - | - | - | - | - | - | - | - | 1 | - | - | 1 |
| Bleeding –  PR | - | - | - | - | 35 | 12 | - | 1 | - | - | - | - | 3 | - | - | 4 | 55 |
| Bowel ischaemia | - | - | - | - | - | - | - | - | - | - | - | - | 1 | - | - | - | 1 |
| Infection –  Intra-abdominal | - | - | - | - | 1 | - | - | - | - | - | - | - | - | - | - | - | 1 |
| Ileus | - | - | - | - | - | - | - | - | - | - | - | 1 | - | - | - | - | 1 |
| Infection –  Chest | 3 | - | - | - | - | - | - | - | - | - | - | - | - | - | - | - | 3 |
| Infection – Wound | 3 | - | - | - | - | - | - | - | - | - | - | - | - | - | - | - | 3 |
| Obstruction | - | - | - | - | - | - | - | - | - | 1 | - | - | - | - | - | - | 1 |
| Pain causing readmission | - | - | - | - | 1 | - | - | - | - | - | - | - | - | - | - | - | 1 |
| Perforation | 11 | - | - | - | - | - | - | - | - | - | - | - | 3 | - | - | - | 14 |
| PPS | 4 | - | - | - | 7 | - | - | - | - | - | - | - | - | - | - | - | 11 |
|  |  |  |  |  |  |  |  |  |  |  |  |  |  |  |  |  |  |
| Combined procedure | **0** | **0** | **0** | **0** | **0** | **0** | **0** | **0** | **0** | **0** | **0** | **0** | **0** | **0** | **2** | **0** | **2** |
| Urinary retention | - | - | - | - | - | - | - | - | - | - | - | - | - | - | 2 | - | 2 |
|  |  |  |  |  |  |  |  |  |  |  |  |  |  |  |  |  |  |
| Surgery –  Trans-anal | **4** | **0** | **0** | **0** | **1** | **0** | **1** | **0** | **1** | **0** | **0** | **0** | **0** | **1** | **4** | **0** | **12** |
| AKI | - | - | - | - | - | - | - | - | 1 | - | - | - | - | - | - | - | 1 |
| Bleeding –  PR | - | - | - | - | 1 | - | 1 | 0 | - | - | - | - | - | - | - | - | 2 |
| Infection –  Intra-abdominal | 1 | - | - | - | - | - | - | - | - | - | - | - | - | - | - | - | 1 |
| Infection –  Chest | 1 | - | - | - | - | - | - | - | - | - | - | - | - | - | - | - | 1 |
| Infection –Wound | 2 | - | - | - | - | - | - | - | - | - | - | - | - | - | - | - | 2 |
| Perforation | - | - | - | - | - | - | - | - | - | - | - | - | - | 1 | - | - | 1 |
| Urinary  retention | - | - | - | - | - | - | - | - | - | - | - | - | - | - | 4 | - | 4 |
|  |  |  |  |  |  |  |  |  |  |  |  |  |  |  |  |  |  |
| Surgery –  Colonic resection | **28** | **2** | **3** | **3** | **4** | **0** | **0** | **0** | **4** | **10** | **1** | **4** | **11** | **7** | **4** | **5** | **86** |
| AKI | - | - | - | - | - | - | - | - | - | 5 | - | - | - | - | - | - | 5 |
| Bleeding –  Intra-abdominal | - | - | - | - | - | - | - | - | - | - | - | - | - | - | - | 1 | 1 |
| Bleeding –  PR | - | - | - | - | 1 | - | - | - | - | - | - | - | - | - | - | 3 | 4 |
| Bowel ischaemia | - | - | - | - | - | - | - | - | - | - | - | - | 1 | - | - | - | 1 |
| DVT/PE | - | 2 | - | - | - | - | - | - | - | - | - | - | - | - | - | - | 2 |
| Haematoma –  Intra-abdominal | 1 | - | - | - | 1 | - | - | - | - | - | - | - | - | 2 | - | 1 | 5 |
| Haematoma –  Wound | 2 | - | - | - | - | - | - | - | - | - | - | - | - | - | - | - | 2 |
| Ileus | - | - | - | - | - | - | - | - | 1 | 5 | - | 4 | - | - | - | - | 10 |
| Infection –  Intra-abdominal | - | - | 1 | - | - | - | - | - | - | - | - | - | - | 1 | - | - | 2 |
| Infection –  Chest | 10 | - | - | - | - | - | - | - | - | - | - | - | - | - | - | - | 10 |
| Infection –  Urine | 4 | - | - | - | - | - | - | - | - | - | - | - | - | - | - | - | 4 |
| Infection –  Wound | 9 | - | - | 3 | - | - | - | - | - | - | - | - | - | 1 | - | - | 13 |
| Leak | 1 | - | 2 | - | - | - | - | - | 1 | - | - | - | 10 | 3 | - | - | 17 |
| Obstruction | 1 | - | - | - | - | - | - | - | - | - | - | - | - | - | - | - | 1 |
| Missing | - | - | - | - | - | - | - | - | 2 | - | 1 | - | - | - | - | - | 3 |
| Pain causing readmission | - | - | - | - | 2 | - | - | - | - | - | - | - | - | - | - | - | 2 |
| Urinary retention | - | - | - | - | - | - | - | - | - | - | - | - | - | - | 4 | - | 4 |
| Total | | | | | | | | | | | | | | | | | **193** |

| 30-DAY READMISSIONS | |  |  |  |
| --- | --- | --- | --- | --- |
|  | **Endoscopy** | **Surgery – Trans-anal** | **Surgery – Colonic resection** | **Total** |
| Related | **55** | **2** | **13** | **70** |
| Bleeding-PR | 40 | 2 | - | 42 |
| Constipation | - | - | 1 | 1 |
| DVT/PE | - | - | 1 | 1 |
| Haematoma – Intra-abdominal | - | - | 1 | 1 |
| Ileus | - | - | 1 | 1 |
| Infection – Intra-abdominal | - | - | 1 | 1 |
| Infection – Urine | - | - | 1 | 1 |
| Infection – Wound | - | - | 3 | 3 |
| Missing | - | - | 2 | 2 |
| Obstruction | 1 | - | - | 1 |
| Pain | 1 | - | 2 | 3 |
| Perforation | 6 | - | - | 6 |
| PPS | 7 | - | - | 7 |
| Unrelated | **12** | **1** | **1** | **14** |

## SUPPLEMENTARY MATERIAL 4 – Complications and reasons for 30-day readmissions

Complete overview of all complications and their treatments divided into procedure type. Values are given as total number. All identified 30-day readmissions after polyp procedures classified into related or unrelated to treatment. The reasons for related readmissions are given as total number. AKI – acute kidney injury, EUA – examination under anaesthetic, DVT – deep vein thrombosis, IR – interventional radiology, ITU – intensive care unit, IV – intravenous, NG – Nasogastric tube, PE – pulmonary embolism, PPS – post polypectomy syndrome PR – per rectum
