# Supplementary material for: Outcomes of complex colorectal polyps managed by multi-disciplinary team strategies—a multi-centre observational study
Source: Int J Colorectal Dis. 2023 Feb 3;38(1):28. doi: 10.1007/s00384-022-04299-0 (PMC9898359; doi:10.1007/s00384-022-04299-0)
Supplement: Supplementary file 5 — Supplementary file5 Final histology (DOCX 30 KB) [file 384_2022_4299_MOESM5_ESM.docx]

|  | Total  (n=1989) | Screening  (n=724) | Non-screening  (n=1265) | *P* value |
| --- | --- | --- | --- | --- |
| Benign | **1814 (91.2%)** | **637 (88.0%)** | **1177 (93.0%)** | *P*<0.001 |
| Adenoma, LGD | 1115 | 376 | 739 |  |
| Adenoma, HGD | 464 | 175 | 289 |  |
| Serrated | 138 | 30 | 108 |  |
| Hyperplastic | 21 | 6 | 15 |  |
| Inflammatory | 10 | 0 | 10 |  |
| Non polyp pathology * | 12 | 6 | 6 |  |
| Histology not available | 54 | 44 | 10 |  |
| Malignant | **175 (8.8%)** | **87 (12.0%)** | **88 (7.0%)** |  |
| Adenocarcinoma | 172 | 85 | 87 |  |
| Other malignancy ** | 3 | 2 | 1 |  |

## SUPPLEMENTARY MATERIAL 5 – Final histology

Values are reported per number of successfully removed lesions and (%) to one decimal place. Comparisons are made between presentations for benign and malignant final histology using a chi-squared test.

* Non polyp pathology included normal mucosa (n=2), lipoma (n=2), anal intraepithelial neoplasia (n=2), papilloma, mucosal prolapse, granulation tissue, fibrosis, fibroepithelial polyp and juvenile polyp (all n=1)

** Other malignancies included neuroendocrine tumour (n=2) and squamous cell carcinoma (n=1)
