# Supplementary material for: Outcomes of complex colorectal polyps managed by multi-disciplinary team strategies—a multi-centre observational study
Source: Int J Colorectal Dis. 2023 Feb 3;38(1):28. doi: 10.1007/s00384-022-04299-0 (PMC9898359; doi:10.1007/s00384-022-04299-0)
Supplement: Supplementary file 6 — Supplementary file6 Characteristics of colonic resections (DOCX 31 KB) [file 384_2022_4299_MOESM6_ESM.docx]

|  | Total  (n=280) | Screening  (n=161) | Non-screening  (n=119) |
| --- | --- | --- | --- |
| Indication for bowel resection |  |  |  |
| Meeting recommendation | 178 (63.6%) | 117 (72.7%) | 61 (51.3%) |
| Unsuccessful endoscopic or combined procedure | 30 (10.7%) | 11 (6.8%) | 19 (16.0%) |
| Cancer suspected during primary procedure | 26 (9.3%) | 14 (8.7%) | 12 (10.1%) |
| Cancer on final histology | 25 (8.9%) | 8 (5.0%) | 17 (14.3%) |
| Residual or recurrent polyp | 14 (5.0%) | 6 (3.7%) | 8 (6.7%) |
| Procedure adverse events | 7 (2.5%) | 5 (3.1%) | 2 (1.7%) |
| Resections performed |  |  |  |
| Right hemicolectomy | 144 (51.4%) | 80 (49.7%) | 64 (53.8%) |
| Anterior resection | 74 (26.4%) | 44 (27.3%) | 30 (25.2%) |
| Sigmoid colectomy | 20 (7.1%) | 17 (10.6%) | 3 (2.5%) |
| Appendicectomy or caecectomy | 14 (5.0%) | 5 (3.1%) | 9 (7.6%) |
| Abdominoperineal resection | 7 (2.5%) | 1 (0.6%) | 6 (5.0%) |
| Left hemicolectomy | 8 (2.9%) | 7 (4.3%) | 1 (0.8%) |
| Subtotal colectomy | 5 (1.8%) | 3 (1.9%) | 2 (1.7%) |
| Hartmann’s procedure | 4 (1.4%) | 2 (1.2%) | 2 (1.7%) |
| Pan proctocolectomy | 4 (1.4%) | 2 (1.2%) | 2 (1.7%) |
| Access |  |  |  |
| Laparoscopic | 210 (75.0%) | 116 (72.0%) | 94 (79.0%) |
| Open | 67 (23.9%) | 42 (26.1%) | 25 (21.0%) |
| Unknown | 3 (1.1%) | 3 (1.9%) | 0 |
| Stoma |  |  |  |
| No | 180 (64.3%) | 91 (56.5%) | 89 (74.8%) |
| Yes – reversed | 19 (6.8%) | 3 (1.9%) | 16 (13.4%) |
| Yes – not reversed | 19 (6.8%) | 5 (3.1%) | 14 (11.8%) |
| Unknown | 62 (22.1%) | 62 (38.5%) | 0 |

## SUPPLEMENTARY MATERIAL 6 – Characteristics of colonic resections

Values are given as number and (%) to one decimal place
